# A review on the inhibitory potential of *Nigella sativa* against pathogenic and toxigenic fungi

**Published:** 2016

**Authors:** Hojjatollah Shokri

**Affiliations:** 1 *Faculty of Veterinary Medicine, Amol University of Special Modern Technologies, Amol, Iran*

**Keywords:** *Nigella sativa*, *Thymoquinone*, *Anti-fungal and anti-aflatoxigenic activity*, *Yeast*, *Dermatophyte*, *Aspergillus*

## Abstract

*Nigella sativa* (*N. sativa*) grows in various parts of the world, particularly in Iran. It has been traditionally used as a folk remedy to treat a number of diseases. The seeds of this plant contain moisture, proteins, carbohydrates, crude fiber, alkaloids, saponins, ash, fixed oils and essential oil. The major components of the essential oil are thymoquinone, p-cymene, trans-anethole, 2-methyl-5(1-methyl ethyl)-Bicyclo[3.1.0]hex-2-en and γ-terpinene. So far, several pharmacological effects such as anti-oxidant, anti-inflammatory, anti-cancer and anti-microbial have been reported for *N. sativa* or its active compounds. Thymoquinone, thymohydroquinone and thymol are the most active constituents which have different beneficial properties. The oil, extracts and some of *N. sativa* active components possessed moderate *in vitro* and *in vivo* inhibitory activity against pathogenic yeasts, dermatophytes, non-dermatophytic filamentous fungi and aflatoxin-producing fungi. The main morphological changes of pathogenic and toxigenic fungi treated with *N. sativa* oil were observed in the cell wall, plasma membrane and membranous organelles, particularly in the nuclei and mitochondria. Although this review represents first step in the search for a new anti-fungal drug, the full potential of *N. sativa *as a fungitoxic agent has not been exploited and necessitates further investigations.

## Introduction

The incidence of both community-acquired and nosocomial fungal infections has significantly increased over the past few decades, accompanying the growing number of high-risk patients, particularly those with impaired immunity (Kauffman, 2006 ▶). The majority of the clinically used antifungals has various drawbacks in terms of toxicity, drug-drug interactions, lack of fungicidal efficacy, cost and emergence of resistant strains caused by the frequent use of some of them (Rapp, 2004 ▶). In spite of the introduction of new anti-fungal drugs, they are still limited in number. Hence, there is a great demand for novel anti-fungal agents, justifying the intense search for new drugs that are more effective and less toxic than those already in use.

Medicinal plants have been used for curing diseases for many countries in different indigenous systems of medicine as well as folk medicine. Essential oils are naturally occurring terpenic mixtures isolated from various parts of plants by steam distillation or other methods (Daferera et al., 2000 ▶). Since these oils are ‘eco-friendly’ and harmless to humans, at present there is an increasing attention, both in industry and academic research, toward medicinal plants and their inhibitory properties against pathogenic and food spoilage fungi (Fan and Chen, 1999 ▶). Essential oils have been empirically used as anti-fungal agents, but the spectrum of activity and mechanisms of action remain unknown for most of them. Although only limited consistent information exists about their activity against human and animals fungal pathogens, some oils have shown important anti-fungal activity against yeasts, dermatophytes and non-dermatophytic filamentous fungi, suggesting their potential therapeutic effect on diseases involving mucosa, the skin and the respiratory tract (Pina-Vaz et al., 2004 ▶; Cavaleiro et al., 2006 ▶; Pinto et al., 2003 ▶). Although oils constitute complementary or alternative therapeutic options that are increasing in popularity, they still have scant scientific credibility. Among various medicinal plants, *Nigella sativa* (*N. sativa*) (family Ranunculaceae) is emerging as a miraculous herb with a rich historical and religious background as many investigators revealed a wide spectrum of pharmacological potential for it (Ali and Blunden, 2003 ▶). *N. sativa* is generally known as black seed and commonly grows in the Middle East, Eastern Europe and Western and Middle Asia. In French it is called nigelle and cumin noir, in German as schwarzkummel, in Italian as nigella, in Spanish as neguilla and pasionara, in Turkish as kolonji, in Hindi as kala zira, in Arabic as Habat-ulSauda and in English as black cumin. Among muslims, *N. sativa* is considered as one of the greatest source of healing medicine available because the black seed is the remedy for all diseases except death, according to prophet Muhammad. It is also recommended for use on regular basis in Tibb-e-Nabawi and identified as the curative black cumin in the Holy Bible, as the Melanthion of Hippocrates and Discroides and as the Gith of Pliny (Junemann, 1998 ▶). Crude extracts and essential oil of* N. sativa* seeds have been reported to possess a number of pharmacological properties such as anti-oxidant (Burits and Bucar, 2000 ▶), anti-tumor (Ivankovic et al., 2006 ▶; Amara et al., 2008 ▶), anti-parasitic (EL Wakil, 2007 ▶), anti-inflammatory (Salem, 2005 ▶; Boskabady et al., 2010 ▶), anti-diabetic (Rchid et al., 2004 ▶), anti-bacterial (Ozmen et al., 2007 ▶; Mariam and Al-Basal, 2009 ▶), anti-fungal effects (Goreja, 2003 ▶; Randhawa and Al-Ghamdi, 2002 ▶; Shigeharu et al., 2006 ▶), protective activity against nephrotoxicity (Uz et al., 2008 ▶) Neurotoxicity (Khazdair, 2015 ▶) and hepatotoxicity (Mahmoud et al., 2002 ▶). This article aims to provide a review of the inhibitory effect of *N. sativa* against pathogenic and aflatoxin-producing fungi as well as describing ultrastructural changes in fungi treated with *N. sativa* oil. 

**Chemical compositions of** ***N. sativa***

Various varieties of* N. sativa* have a range of phytochemical components, of which only some molecules were characterized; so, complementary investigations are needed to identify new compounds in this species. Up to now, many investigations have been done on the seeds of *N. sativa* (Table 1). The main compounds were proteins, carbohydrates, fixed oils, essential oil, crude fiber, alkaloids, minerals, vitamins, ash and moisture. Other components were tannins, resin, saponin, carotene, glucosides and sterols (Randhawa and Al-ghamdi, 2002 ▶). α-sitosterol was a major sterol accounting for 44% and 54% of the total sterols in Tunisian and Iranian varieties of black seed oils, respectively (Cheikh-Rouhou et al., 2008 ▶). Selenium, DL-α-tocopherol, DL-γ-tocopherol, all trans retinol were among important anti-oxidants present in *N. sativa *seeds (Nasir et al., 2005 ▶; AL Saleh et al., 2006 ▶). Root and shoot were reported to contain vanillic acid (Al-Jassir, 1992 ▶). 

**Table 1:** Nutrient contents of *N. sativa*

**Nutrient** **s**	**%**	**Compositions**	**References**
**Moisture**	4	-	Khan et al., 2003; El-Tahir and Bakheet, 2007
**Total protein**	20-22	arginine, glutamic acid, leucine, lysine, methionine, tyrosine, proline, threonine, etc.	Khan et al., 2003; El-Tahir and Bakheet, 2007
**Total carbohydrate**	17-34	-	Takruri and Dameh, 1998; Atta, 2003
**Minerals**	1.79-3.74	calcium, copper, iron, zinc, potassium, magnesium, phosphorus, sodium, manganese	Sultan et al., 2009; Youssef et al., 2013
**Alkaloids**	2.06	nigellicine, nigeledine, nigellimine	Atta-Ur-Rahman, 1995
**Fixed oil**	31-36	linoleic acid, oleic acid, palmitic acid, palmitoleic acid, myristic acid, stearic acid, linolenic acid, arachidic acid, lauric acid, eicosadienoic acid	Staphylakis and Gegiou, 1986; Asdadi et al., 2014; Nickavar et al., 2003
**Essential oil**	0.5-1.6	-	Zaoui et al., 2002; Morikawa et al., 2004; Morikawa et al., 2004; Ali et al., 2008; Mehta et al., 2009
**Crude fiber**	8-8.4	-	Khan et al., 2003; El-Tahir and Bakheet, 2007
**Ash**	4.5-4.8	-	Khan et al., 2003; El-Tahir and Bakheet, 2007
**Vitamins**	0.3	B_1_, B_2_, B_6_, niacin, folic acid	Nergiz and Otles, 1993

The major components of the essential oil are thymoquinone (Venkatachallam et al., 2010 ▶; Mahmoudvand et al., 2014 ▶), p-cymene (Sultan et al., 2009 ▶; Toma et al., 2010 ▶; Sunita and Meenakshi, 2013 ▶), trans-anethole (Gerige et al., 2009 ▶; Shokri et al., 2012 ▶), 2-methyl-5-(1-methyl ethyl)-Bicyclo[3.1.0]hex-2-en (Adamu et al., 2010 ▶) and γ-terpinene (Golparvar et al., 2013 ▶). Some previous studies reported other chemical components including limonene, α-thujene, α-pinene, β-pinene, thymol, carvacrol, nigellone, anisaldehyde, n-nonane, miristicine, camphene, β-myrcene, 1,8-cineole, longipinene, camphor, linalool, estragole, junipene, germacrene, aromadendrene, borneol, bornylacetat, 2-tridecanone, 4-terpineol, sabinene, phencen, apiol, carvene, carvone, caryophyllene, avenasterol-5-ene, avenasterol-7-ene, campestrol, cholesterol, citrostadienol, cycloeucalenol, gramisterol, lophenol, obtusifoliol, longifolene, stigmastanol, stigmasterol-7-ene, β-amyrin, butyrospermol, cycloartenol, 24-methylene-cycloartanol, taraxerol, tirucallol, 3-O-[β-D-xylopyranosyl (1→3)-α-L-hamnopyranosyl (1→2)-α-L-arabino-pyranosyl]-28-O-[α-L-rhamnopyranosyl (1→4)-β-D-glucopyranosyl (1→6)-β-D-gluco-pyranosyl] hederagenin, aliphatic alcohol, β-unsaturated hydroxy ketone, melanthin, melanthigenin, 3-O-[β-D-xylopyranosyl-(1→2)-α-L-rhamno-pyranosyl-(1→2)-β-D- glucopyranosyl]-11-ethoxy-6, 23-dihydroxy-28-methy-lolean-12-enoate, stigma-5, 22-dien-3-β-D-gluco-pyranoside and cycloart-triene-23-ethyl-7 (Zaoui et al., 2000 ▶; Morikawa et al., 2004 ▶; Ali et al., 2008 ▶; Mehta et al., 2009 ▶).

**Inhibitory effect of** ***N. sativa*** **against pathogenic yeasts**

Candidiasis caused by *Candida* species has increased dramatically in recent years. *Candida* is the third- or fourth-most-common isolate in nosocomial bloodstream infections in the world. Among various species, *Candida albicans* (*C. albicans*) is the most causative agent associated with serious fungal infection, accounting for more than 90% of cases (Douglas, 2003 ▶).The difficulties associated with the management of *Candida* infections necessitate the discovery of new anti-fungal agents in order to increase the spectrum of activity against* Candida *and combat strains showing resistance to the available anti-fungal drugs. According to the literature, the investigation of natural products activity against *Candida *species increased significantly in the last 10 years, focusing on investigation of approximately 258 plant species from 94 families (Feldmesser, 2003 ▶; Duarte and Figueira, 2008 ▶). Table 2 summarizes the effect of *N. sativa* against different pathogenic yeasts as assessed by standard susceptibility methods. In general, a moderate efficacy of the oil from *N. sativa* against different *Candida* species were demonstrated (Naeini et al., 2009 ▶; Shokri et al., 2012 ▶; Asdadi et al., 2014 ▶)*.* In addition, several studies demonstrated that methanolic extract of *N. sativa* has the strongest anti-fungal effect against different strains of pathogenic yeasts, followed by ethanolic and chloroform extracts (Raval et al., 2010 ▶; Ahmad et al., 2013 ▶). 

**Table 2:** Antifungal activity of *N. sativa* against different pathogenic yeasts

**References**	**Growth Inhibition **	**Susceptibility method**	**Type**	**Fungus**
Naeini et al., 2009	IZ*: 35 mm**	Disc diffusion	Essential oil	***Candida albicans***
Naeini et al., 2009	MIC: 2300 µg/ml	Broth macrodilution	Essential oil	***C. albicans***
Shokri et al., 2012	IZ: 40.8 mm	Disc diffusion	Essential oil	***C. zeylanoides***
Asdadi et al., 2014	MIC_90_: 4.916 mg/ml MFC: 6.360 mg/ml	Broth macrodilution	Essential oil	***C. albicans***
Asdadi et al., 2014	MIC_90_: 5.183 mg/ml MFC: 6.360 mg/ml	Broth macrodilution	Essential oil	***C. dubliniensis***
Asdadi et al., 2014	MIC_90_: 5.992 mg/ml MFC: 6.360 mg/ml	Broth macrodilution	Essential oil	***C. glabrata***
Asdadi et al., 2014	MIC_90_: 4.939 mg/ml MFC: 6.360 mg/ml	Broth macrodilution	Essential oil	***C.krusei***
Raval et al., 2010	MIC: 4.846 µg/ml	Broth microdilution	Methanolic extract	***C. parapsilosis***
Raval et al., 2010	MIC: 6.484 µg/ml	Broth microdilution	Methanolic extract	***C. albicans***
Raval et al., 2010	MIC: 6.795 µg/ml	Broth microdilution	Methanolic extract	***Issatchenkiaorientalis***
Raval et al., 2010	MIC: 5.805 µg/ml	Broth microdilution	Ethanolic extract	***I. orientalis***
Raval et al., 2010	MIC: 7.093 µg/ml	Broth microdilution	Ethanolic extract	***C. parapsilosis***

* IZ: Inhibition zone,

** mm: millimeter

In an experimental study by Khan et al. (2003) ▶, the aqueous extract of *N. sativa* seed exhibited inhibitory effect against candidiasis in mice. A 5-fold decrease in* Candida* organisms in kidneys, 8-fold in liver and 11-fold in spleen was observed in the groups of animals post-treated with the plant extract. It has been shown that the candidacidal pathway in mice neutrophils is nitric oxide (NO)-dependent (Fierro and Fidalgo, 1996 ▶). It is possible that the plant extract contains active ingredient(s), which may directly stimulate the granulocytes and monocytes to generate NO leading to an excellent anti-fungal activity, which in turn kills *C. albicans*. One of the anti-fungal actions of *Nigella* seed’s oil may be attributed to the presence of β-sitosterol and oleic acid as the main components in the oil of *N. sativa* (Asdadi et al., 2014 ▶)*.* Several previous studies have shown that long-chain fatty acid has a fungistatic effect against a few strains of *Candida* (Ouraïni et al., 2007 ▶). In addition, Gupta et al. (2012) ▶ exhibited that different components of *N. sativa* oil such as β-sitosterol and stigmasterol have anti-fungal activity against pathogenic yeasts such as *Candida tropicalis, C. albicans* and *Geotrichum candidum*. In a study by Sitheeque et al. (2009) ▶, all the polyphenols of black seed had anti-*Candida* activity against all tested *Candida* species such as *C. albicans* and *C. glabrata*, followed by *C. parapsilosis*, *C. krusei* and *C. tropicalis*.

The anti-yeast activity of quinones such as dithymoquinone and thymohydroquinone from *N. sativa* were evaluated *in vitro* using a broth microdilution method against six dairy spoilage yeast species. It is found that anti-fungal effects of quinones were comparable with those of preservatives commonly used in milk products (calcium propionate, natamycine and potassium sorbate), while thymohydroquinone possessed significant anti-yeast activity (Halamova et al., 2010 ▶). As reported by Taha et al. (2010) ▶, the minimum inhibitory concentration (MIC) of thymol was 0.5 mg/ml for *C. albicans*, *Rhodoturula rubra* (*R. rubra*) and *Trichosporon* species. *R. rubra* was the most sensitive yeast to thymoquinone (0.1 mg/ml), followed by *Trichosporum* species (0.25 mg/ml) and *C. albicans* (the resistant yeast). The MIC of thymohydroquinone was 0.5 mg/ml for all yeasts except *R. rubra,* which was inhibited at 0.25 mg/ml. In general, thymoquinone was the most potent active compound of *N. sativa* oil against the yeasts, followed by thymohydroquinone and thymol, respectively. In a comparative study on anti-fungal activity of tannins, saponins, alkaloids, essential oil, brown and yellow crystals separated from essential oil, all tested *N. sativa* constituents except for tannins showed inhibitory activity against *Saccharomyces cerevisiae* (Shohayeb and Halawani, 2012 ▶). 

**Inhibitory effect of** ***N. sativa*** **against dermatophytic fungi**

One of the most important groups of fungi, which causes worldwide human and animal infections is the dermatophytes. They have the ability to invade keratinized tissues such as hair, skin and nails to produce an infection, commonly referred to as dermatophytosis (Sidat et al., 2006 ▶). At present, there are many anti-dermatophytic drugs including imidazoles and terbinafine for topical, and triazoles and griseofulvin for systemic treatment of dermatophytosis, which exhibited some problems such as prolonged systemic therapy, fungal resistance, high toxicity and high cost (Martinez-Rossi et al., 2008 ▶). For these reasons, development of new drugs or combination therapy for treatment of dermatophytosis is urgent. Various studies have shown that plant extracts and plant-derived components are effective against dermatophytosis. Table 3 exhibited the efficacy of *N. sativa* against different dermatophytes by using standard susceptibility methods. Several studies showed a weak to moderate inhibitory effect against a considerable number of clinical isolates of various dermatophytes (Aljabre et al., 2005 ▶; Gerige et al., 2009 ▶; Khosravi et al., 2013 ▶; Sunita and Meenakshi, 2013 ▶; Mahmoudvand et al., 2014 ▶).

**Table 3:** Antifungal activity of *N. sativa* against different dermatophytes

**References**	**Growth inhibition **	**Susceptibility method**	**Type **	**Fungus**
Khosravi et al., 2013	MIC: 2±0.6 mg/ml MFC: 4±1.1 mg/ml	Broth microdilution	Essential oil	***Trichophyton mentagrophytes***
Khosravi et al., 2013	MIC: 4±1.1 mg/ml MFC: 4±1.1 mg/ml	Broth microdilution	Essential oil	***Trichophyton rubrum***
Khosravi et al., 2013	MIC: 2±0.6 mg/ml MFC: 4±1.1 mg/ml	Broth microdilution	Essential oil	***Epidermophyton floccosum***
Khosravi et al., 2013	MIC: 2±0.6 mg/ml MFC: 4±1.1 mg/ml	Broth microdilution	Essential oil	***Microsporum gypseum***
Khosravi et al., 2013	MIC: 4±1.1 mg/ml MFC: 8±2.8 mg/ml	Broth microdilution	Essential oil	***Microsporum canis***
Gerige et al., 2009	IZ*: 0.0 mm** (resistant)	Disc diffusion	Essential oil	***Trichophyton mentagrophytes***
Aljabre et al., 2005	MIC: 40 mg/ml	Agar diffusion	Ether extract	***Trichophyton mentagrophytes***
Aljabre et al., 2005	MIC: 40 mg/ml	Agar diffusion	Ether extract	***Trichophyton interdigitale***
Aljabre et al., 2005	MIC: 40 mg/ml	Agar diffusion	Ether extract	***Trichophyton rubrum***
Aljabre et al., 2005	MIC: 10 mg/ml	Agar diffusion	Ether extract	***Microsporum canis***
Aljabre et al., 2005	MIC: 40 mg/ml	Agar diffusion	Ether extract	***Epidermophyton floccosum***
Mahmoudvand et al., 2014	MIC: 4 mg/ml MIC: 8 mg/ml MIC: 16 mg/ml	Broth macrodilution	Essential oil Methanolic extract Aqueous extract	***Trichophyton mentagrophytes***
Mahmoudvand et al., 2014	MIC: 4 mg/ml MIC: 4 mg/ml MIC: 8 mg/ml	Broth macrodilution	Essential oil Methanolic extract Aqueous extract	***Microsporum canis***
Mahmoudvand et al., 2014	MIC: 4 mg/ml MIC: 8 mg/ml MIC: 16 mg/ml	Broth macrodilution	Essential oil Methanolic extract Aqueous extract	***Microsporum gypseum***
Sunita and Meenakshi, 2013	IZ: 38 mm	Disc diffusion	Essential oil	***Microsporum gypseum***
Sunita and Meenakshi, 2013	IZ: 20 mm	Disc diffusion	Essential oil	***Trichophyton rubrum***
Sunita and Meenakshi, 2013	IZ: 35 mm	Disc diffusion	Essential oil	***Trichophyton simii***

* IZ: Inhibition zone,

** mm: millimeter

As reported by Taha et al. (2010), thymol was found to be effective against *Trichophyton violaceum* (*T. violaceum*) (MIC 0.1 mg/ml), *Microsporum canis* (*M. canis*) (MIC 0.1 mg/ml) and *T. mentagrophytes* (MIC 0.05 mg/ml). The MIC of thymoquinone was found to be 0.05 mg/ml for tested dermatophytes. The MIC of thymohydroquinone was found to be 0.1 mg/ml for *M. canis*, while it showed various values (from 0.025 to 0.25 mg/ml) for *T. mentagrophytes* isolates. In general, thymoquinone was more efficient against the dermatophytes, followed by thymohydroquinone and thymol, respectively. Similarly, Ali and Blunden (2003) ▶ indicated that most of biological activity of the *N. sativa* seeds was due to thymoquinone, the major component of the essential oil obtained using soxhlet extraction. Thus, high anti-dermatophytic activity of *N. sativa* oil could be due to higher content of thymoquinone in comparison with methanolic and aqueous extracts, as previously described (Aljabre et al., 2005 ▶). Geweely and Alakilli (2012) ▶ isolated a variety of anti-fungal proteins from *N. sativa*. The crude proteins of *N. sativa* had highly significant anti-dermatophytic activity on four zoophilic dermatophytes (*M. canis*, *M. equinum*, *T. mentagrophytes* and *T. verrucosum*). *N. sativa* proteins had considerable effects on the fungal cell permeability of all zoophilic dermatophytes. Two purified proteins (Pr1 and Pr2) from* N. sativa* showed higher anti-dermatophytic activities, representing 1.43-fold of the crude protein. The above-mentioned results denote the potential efficacy of *N. sativa* as a source of anti-dermatophytic drugs and support its use in folk medicine for the treatment of fungal infections.

**Inhibitory effect of** ***N. sativa*** **against non-dermatophytic filamentous fungi**

Literature survey showed that there is not enough research about anti-fungal activity of *N. sativa* against non-dermatophytic fungi. Table 4 exhibited the effect of *N. sativa* against different non-dermatophytic filamentous fungi by standard susceptibility methods. A relatively moderate activity of the oil and extracts of *N. sativa* against filamentous fungi was demonstrated (Sitara et al., 2008 ▶; Khosravi et al., 2011 ▶; Singh et al., 2015 ▶)*.*

**Table 4:** Antifungal activity of *N. sativa* against non-dermatophytic filamentous fungi

**References**	**Growth inhibition **	**Susceptibility method**	** Type**	**Fungus**
Khosravi et al., 2011	MIC_90_: 1.5 mg/ml MIC_90_: 1.5 mg/ml	Broth macrodilution Broth microdilution	Essential oil	***Aspergillus fumigatus***
Khosravi et al., 2011	MIC_90_: 1.5 mg/ml MIC_90_: 2 mg/ml	Broth macrodilution Broth microdilution	Essential oil	***A.flavus***
Sitara et al., 2008	IZ*: 0.33 mm**	Agar diffusion	Essential oil	***A.niger***
Sitara et al., 2008	IZ: 0.16 mm	Agar diffusion	Essential oil	***A.flavus***
Sitara et al., 2008	IZ: 0.0 mm	Agar diffusion	Essential oil	***Fusarium moniliforme***
Sitara et al., 2008	IZ: 0.06 mm	Agar diffusion	Essential oil	***F. oxysporum***
Sitara et al., 2008	IZ: 0.260 mm	Agar diffusion	Essential oil	***F. nivale***
Sitara et al., 2008	IZ: 0.0 mm	Agar diffusion	Essential oil	***F. semitectum***
Sitara et al., 2008	IZ: 0.0 mm	Agar diffusion	Essential oil	***Drechslera hawiensis***
Sitara et al., 2008	IZ: 0.0 mm	Agar diffusion	Essential oil	***Alternaria alternata***
Singh et al., 2015	IZ: 45.7 mm IZ: 8.9 mm	Inverted petri plate	Essential oil Ethanolic extract	***A. ﬂavus***
Singh et al., 2015	IZ: 43.6 mm IZ: 5.7 mm	Inverted petri plate	Essential oil Ethanolic extract	***A.niger***
Singh et al., 2015	IZ: 39.7 mm IZ: 10 mm	Inverted petri plate	Essential oil Ethanolic extract	***F. graminearum***
Singh et al., 2015	IZ: 71.2 mm IZ: 17.8 mm	Inverted petri plate	Essential oil Ethanolic extract	***F. moniliforme***
Singh et al., 2015	IZ: 34.7 mm IZ: 9.9 mm	Inverted petri plate	Essential oil Ethanolic extract	***Penicillium viridicatum***

* IZ: Inhibition zone,

** mm: millimeter

In a study conducted by Taha et al. (2010), the various MICs of thymol were shown. *Fusarium oxysporum* (*F. oxysporum*) and *Cladosporium* species were the most sensitive isolates, which were inhibited at 0.1 mg/ml, whereas *Aspergillus niger* (*A. niger*) and *Penicillium chrysogenum* (*P. chrysogenum*) were the most resistant, which were inhibited at 0.5 mg/ml. The MIC of thymoquinone showed similar profile as that obtained by thymol. *F. oxysporum* (MIC 0.1 mg/ml) and *A. niger* (MIC 0.1 mg/ml) together with *P. chrysogenum* (MIC 0.75 mg/ml) were the most sensitive and resistant isolates, respectively. *Cladosporium* species were inhibited at 0.25 mg/ml. The same profile that was obtained by thymol was also obtained by thymohydroquinone. *F. oxysporum* and *Cladosporium* species were the most sensitive organisms to thymohydroquinone. Both of them were inhibited at 0.25 mg/ml. *A. niger* and *P. chrysogenum* were the most resistant organisms (MIC 0.75 mg/ml). In general, thymol was the most efficient chemical against the tested filamentous fungi, followed by thymoquinone and thymohydroquinone, respectively. In another study, two novel anti-fungal defensins, namely Ns-D1 and Ns-D2, were isolated from seeds of *N. sativa*. Both defensins displayed strong divergent anti-fungal activity towards a number of phtopathogenic fungi (Rogozhin et al., 2011 ▶).

**Inhibition of aflatoxin production by** ***N. sativa***

Mycotoxin-producing fungi are important contaminants and destroyers of food and feedstuffs during the storage, rendering them inappropriate for human consumption by reducing their nutritive value and sometimes by producing mycotoxins (Kumar et al., 2007 ▶). Mycotoxins are toxic to human and animals, and cause significant reductions in crop yield resulting in economic loss (Iheshiulor et al., 2011 ▶). The food and agriculture organization of the United Nations (FAO) estimated that at least 25% of the cereal grains in the world are contaminated by mycotoxins like aflatoxins (Bathnagar and Garcia, 2001 ▶). Beside synthetic agents, some plants and their active metabolites have been introduced as inhibitors of aflatoxin biosynthesis (Sakuda et al., 2000 ▶; Rasooli and Razzaghi-Abyaneh, 2004 ▶; Yoshinari et al., 2007 ▶; Roze et al., 2011 ▶). To our knowledge, little data have been reported on the effect of *N. sativa* against aflatoxin-producing fungi. In a study by Khosravi et al. (2011), the effect of the essential oil of *N. sativa* on growth and aflatoxin production of *A. parasiticus* was evaluated. The results indicated that *N. sativa* oil (MIC_90_ 2.75 mg/ml and MFC 6.25 mg/ml) had a moderate activity against *A. parasiticus*. Essential oil of *N. sativa* (1.5 mg/ml) exhibited a growth inhibition percent of mycelia production by *A. parasiticus* in value of 67.4%. In addition, aflatoxin production was inhibited by 1.5 mg/ml of *N. sativa* oil, representing significant reductions in values of 91.4% for AFB_1_, 100% for AFB_2_, 98.5% for AFG_1_, 87.3% for AFG_2_ and 96.2% for total aflatoxin. The oil had a more marked effect on aflatoxin B_2 _as compared toAFB_1_. This study showed that the extent of inhibition in mycelial growth was associated with decreased levels of aflatoxin production. Interestingly, aflatoxin production was significantly inhibited at concentrations lower than fungistatic concentrations of the oil. There is a 10-kb gene cluster which controls the activity of aflatoxins biosynthesis pathway. Regarding different aflatoxins, it seems that some of the genes are more active than the others. The correlations between some gene expressions and production of different kinds of aflatoxins have been previously noted. It is speculated that the essential oil of *N. sativa* may affect the expression of some special aflatoxin gene (Yahyaraeyat et al., 2013 ▶). Further studies are needed to evaluate this issue. In a study by Maraqa et al. (2007) ▶, the crude extract of *N. sativa* inhibited the production of three types of aflatoxins (B_1_, B_2_ and G_1_) at 5% (w/v) concentration, whereas *N. sativa* oil at a concentration of 3% completely inhibited aflatoxins B_1_, B_2_, G_1_ and G_2_. The authors recommended that the anti-aflatoxin property of the oil is mainly related to its high phenolic content as demonstrated previously (Cosentino et al., 1999 ▶). In a similar study conducted by El-Nagerabi et al. (2012) ▶, *N. sativa* oil at a concentration of 3% significantly inhibited aflatoxin B_1_ production by *A*. *flavus *and *A. parasiticus *by 47.9-58.3% and 32-48%, respectively. On the other hand, the mycelial fresh weights of the two fungal species were similar at the tested concentrations. *N. sativa* oil 3% against pure aqueous aflatoxin B_1_ led to decrease of the concentration to 670 ppb in comparison with the control (690 ppb), representing no detoxification ability on aflatoxin B_1_. Moreover, Al-Ghasham et al. (2008) ▶ demonstrated that *N. sativa* reduces the toxic effect of AFB_1_ in liver and kidneys of rats with aflatoxicosis, which may be related to their cytoprotective and anti-oxidant properties. Recently, Abdel-Wahhab and Aly (2005) ▶ found that *N. sativa* oil administration to rats fed with aflatoxin-contaminated diet resulted in significant protection against aflatoxicosis. Similarly, Hussein et al. (2000) ▶ found that dietary addition of *N. sativa* significantly ameliorated the adverse effects of dietary AFB_1_ on Nile tilapia fish. To the best of our knowledge, there were no previous studies exploring the effect of *N. sativa* on other toxins produced by toxigenic fungi.

**Ultrastructural changes of pathogenic and toxigenic fungi due to** ***N. sativa***

**Figure 1:**
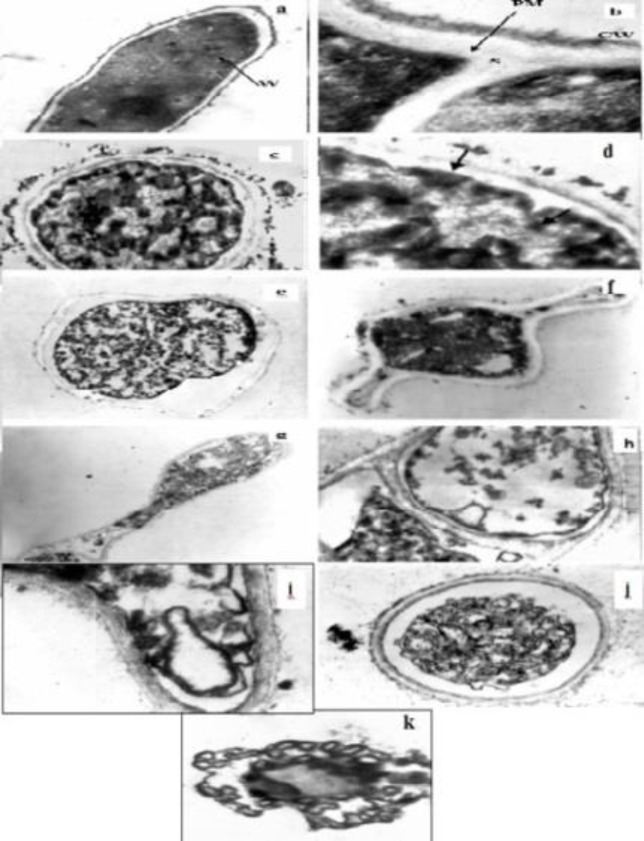
Transmission electron micrographs of *Aspergillus* species (**CW:** Cell wall; **PM:** Plasma membrane; **S:** Septum; **W:**Woronin body

 As mentioned above, various studies have been carried out to investigate the anti-fungal activity of *N. sativa*, but its exact mechanism of action was not well established. Furthermore, very few data have been documented on the morphological changes of pathogenic and/or toxigenic fungi grown in the presence of *N. sativa* oil and its components. In a study conducted by Khosravi et al. (2011) ▶, *A. flavus* (toxigen) and *A. fumigates* (pathogen) exposed to 0.25, 0.5, 1, 1.5 and 2 mg/ml of *N. sativa* essential oil were processed for transmission electron microscopy (TEM) (Figures 1a and 1b; normal shapes). The early changes in fungal compartments in the presence of the lowest concentrations of oil (0.25 and 0.5 mg/ml) were noticed in both hyphae and conidia, showing abnormal shaped and swelled hyphae, high vacuolation of the cytoplasm accompanied by vacuole fusion (Figures 1c and 1d). Subsequent events were loss of normal conidia and hyphae shape, detachment of fibrillar layer of the cell wall (Figures 1e, 1f and 1g), destruction of hypha memberanous organelles including nuclei and mitochondria, and finally disorganization of cytoplasmic contents accompanied by intensive degradation and lysis of the nucleus and mitochondria (Figure 1h). The most remarkable changes in fungal compartments were observed in fungi treated with the highest fungistatic concentrations of the oils (1.5-2 mg/ml). Destruction and breaking down of plasma membrane at different sites (Figure 1i), disorganization of conidial and hyphal cytoplasm and complete lysis of membranous organelles seemed to be resulting in cells death (Figures 1j and 1k).

All together, the main changes were in the cell wall, plasma membrane and membranous organelles; in particular, in the nuclei and mitochondria. Interestingly, Badary et al. (2000) ▶ indicated that adding of thymoquinone as the main component of *N. sativa* seed to the drinking water of mice at a concentration of 0.03% for 3 months led to no sign of toxicity, except for a significant decrease in fasting plasma glucose concentration. Therefore, the *N. sativa* seeds and their derivatives appear to have a low level of toxicity and could be considered safe at low concentrations in the host cells.

The use of herbal drugs as complementary medicine is prevalent and has gained worldwide popularity. Many drugs are derived directly from plants; while others are chemically modified natural products. The original research articles published so far have confirmed the anti-fungal potential of *N. sativa* seeds. The oil, extracts and some of its active components, particularly thymoquinone, thymohydroquinone and thymol, possess moderate *in vitro* and *in vivo* inhibitory effect against pathogenic yeasts, dermatophytes, non-dermatophytic filamentous fungi and aflatoxin-producing fungi. 
